# Periprosthetic and peri‐implant femoral fractures and timeliness to surgery: A retrospective matched cohort study

**DOI:** 10.1002/jeo2.70037

**Published:** 2024-10-16

**Authors:** Felix Alarcón, Olof Sköldenberg, Martin Magnéli, Michael Axenhus

**Affiliations:** ^1^ Department of Clinical Sciences at Danderyd Hospital, Unit of Orthopaedics Karolinska Institutet Stockholm Sweden; ^2^ Department of Orthopaedic Surgery Danderyd Hospital Stockholm Sweden

**Keywords:** adverse events, delay to surgery, periprosthetic femoral fractures, retrospective cohort study, time to surgery

## Abstract

**Purpose:**

Periprosthetic (PPFF) and peri‐implant femoral fractures (PIFFs) are troublesome complications of prosthetic and implant surgery, the prior being described to have a greater delay to surgery when compared with standard hip fractures. The implications of PPFF delay being disputed in the current literature and those of PIFF have not been investigated. The aim of this study was to determine whether the time from radiological examination to surgery differs between hip fractures and PPFF/PIFF, and the possible consequences of delay and group affiliation on morbidity, mortality, and readmissions.

**Methods:**

One hundred and thirty‐six participants were admitted to Danderyd hospital during 2020, cases exposed to PPFF or PIFF (*n* = 35) and hip fracture controls (*n* = 101) matched at 1:3 with respect to age and sex. Timestamps from radiology, surgery, and death were retrieved from the Swedish fracture registry, data on adverse events (AEs), and readmissions were collected through retrospective medical record review for 90‐days postsurgery.

**Results:**

Linear regression showed that time to surgery differed in case and control cohorts by a mean of 24.8 h, *p* < 0.001, and AEs were significantly more common in cases, *p* = 0.046. Unadjusted binary logistic regression indicated a possible relationship between time to surgery increasing the rate of AEs by 1.3% per hour of delay, 95% confidence interval [CI]: (1–1.03).

**Conclusion:**

This study reveals a significant delay in surgery for PPFFs and PIFFs compared with standard hip fractures, leading to higher adverse event rates. While mortality and readmissions did not differ significantly, the delay underscores the need for timely intervention in these complex cases. Further research is needed to address these challenges and improve patient outcomes.

**Level of Evidence:**

III

## Graphical abstract

Delays in surgery for periprosthetic (PPFF) and peri‐implant femoral fractures (PIFF) are a serious clinical concern. This study found that PPFF and PIFF patients experienced a significant delay in surgery, which correlated with higher adverse event rates.

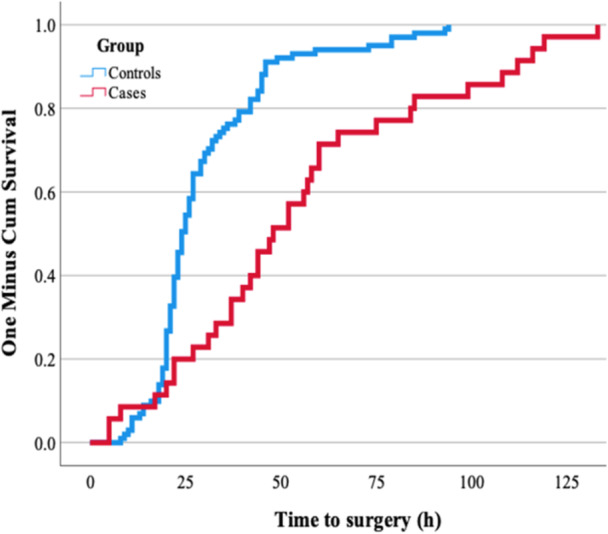

**Abbreviations**
AE: adverse events
AO/OTA: AO Foundation and Orthopaedic Trauma Association
ASA: American Society of Anaesthesiologists
BMI: body mass index
CFS: Clinical Frailty Scale
CI: confidence interval
NOMESCO: Nordic Medico‐Statistical Committee
PIFF: peri‐implant femoral fracture
PPFF: periprosthetic femoral fracture
REDCap: Research Electronic Data Capture
SFR: Swedish Fracture Registry
THA: total hip arthroplasties
UCS: Unified Classification System
VCS: Vancouver Classification System

## INTRODUCTION

Fractures of the hip are a common occurrence in Scandinavia with the highest rates of proximal femoral fractures in the world [3]. Sweden in particular has a yearly hip fracture incidence of 709/100,000 in women and 303/100,000 in men [5]. Trends show incidence increasing with age, due to factors such as decreased bone mineral density and reduced levels of activity with women being particularly vulnerable to hip fractures [4, 18]. Hip fractures are generally classified according to a compendium produced by the AO foundation and orthopaedic trauma association (AO/OTA classification) [26].

Periprosthetic femoral fractures (PPFFs) are complications following prosthetic hip surgery and a growing issue due to the increasing amount of total hip arthroplasties (THAs) performed [2]. Additionally, the increasing longevity of patients and associated comorbidities such as osteoporosis have been observed to impact the occurrence of PPFF [23, 24]. The mortality of these patients parallels and, in some cases, exceeds that of hip fracture arthroplasties [8, 10, 25]. In more recent times, PPFFs are classified according to the Unified Classification System (UCS), a development of the Vancouver classification system (VCS) [20]. A relatively unstudied field, peri‐implant femoral fractures (PIFFs) are serious complications to internal fixations in hip fracture surgery. Arising in close proximity to nonprosthetic implants such as cephalomedullary nails and sliding hip devices.

Time to surgery is an important measure for hip fractures as delays are associated with increased risk of adverse events (AEs), mortality, and postoperative complications [14, 22]. The Swedish hip fast‐track as a process in which the patient upon arrival is prepared for surgical intervention within 24 h from admission [6]. Early surgery, within 48 h, is correlated with decreased 1‐year mortality and fewer perioperative complications [15]. As of yet, there are no studies observing the effects of surgical delay in PPFF/PIFF.

Existing literature presents conflicting evidence regarding the impact of such delays on morbidity and mortality. Some studies have found that delays to surgery of between 24 and 72 h to be significant for an increased risk of an AE [1, 8]. However, similar studies have found that there is no significant difference in outcomes regarding delay to surgery [12, 21]. While the importance of prompt surgery for standard hip fractures is well established, the implications of surgical delays for PPFFs and PIFFs remain unclear. Furthermore, the PIFF and PPFF population is only expected to increase in size in the coming years due to the increase in the elderly population. Lastly, the effects of delays on the relatively unstudied PIFFs have not been investigated at all.

The primary aim of this study was to investigate whether the time from radiological examination to surgery differs between patients with standard hip fractures and those with PPFFs and PIFFs. A secondary aim was to explore the potential consequences of these delays on morbidity, mortality, and readmissions within 90 days postsurgery.

## MATERIALS AND METHODS

### Study design and setting

This is a single‐center matched cohort study in Danderyd University hospital, Sweden. The hospital has a catchment population of approximately 700,000 in the greater Stockholm area. Data collection was performed through medical record review and retrieval of data from the Swedish fracture registry (SFR) from the inclusion period, 01 January 2020 to 31 December 2020 [19]. We chose a follow‐up duration of 1 year as the most significant postoperative events, including mortality and readmissions, occuring within this timeframe. Follow‐up were conducted up to 90 days postsurgery through reviewing patient medical records for AEs, deaths, and readmissions to inpatient care.

### Data source

SFR prospectively collects data including age at injury, sex, fracture classification according to AO/OTA, UCS and PIFF, mechanism of injury, method of surgical intervention and timestamps from radiology, as well as time of surgery and reoperation. The outcome variables retrieved include death, as well as variables calculated from timestamps such as time to surgery and time to reoperation. Data assembly was performed using Research Electronic Data Capture (REDCap) [9].

### Participants

All patients admitted to Danderyd hospital during 2020 for hip fractures and PPFF/PIFF were considered potential participants as described by inclusion and exclusion criteria (Table 1).

**Table 1:** Inclusion and exclusion criteria for the observational cohort.

Inclusion	Exclusion
Patients ≥50 years of age.	Atypical fracture as registered in SFR.
Periprosthetic & peri‐implant fractures ICD‐code M96.6 F or as registered in SFR.	ICD‐code for Pathological fracture M84.4
Hip fractures: collum femoris, per‐ and subtrochanteric fractures, ICD‐code S72.	Radiological examination was performed outside of Danderyd hospital.
Hip fractures requiring any form of surgical intervention.	MRT necessary for diagnosis, exclusion through reviewing medical records.
Admission and surgery at Danderyd hospital during 2020.	Missing data necessary for the analysis of primary outcomes.

Abbreviations: ICD, International Classification of Diseases; SFR, Swedish Fracture Registry.

We excluded atypical and pathological fractures, which require different considerations when preparing/undergoing surgery. Additionally, patients having undergone radiological examination at another facility or required magnetic resonance tomography to provide a diagnosis were excluded due to this group having a longer delay from injury to diagnosis.

#### Matching

The matching was conducted by a clinician impartial to the project and made from the 35 patients in the case cohort. Matching was made with respect to age and sex at a 3:1, control‐to‐case ratio, where the accepted age range for matching was +/– 3 years.

#### Baseline variables

Baseline variables were retrieved from SFR. Sex, age, American Society of Anaesthesiologists (ASA)‐ class, body mass index (BMI), Clinical Frailty Scale (CFS), and cognitive impairment status were collected. Fracture classes and surgical methods and surgeon experience level were retrieved from SFR, due to the lack of classification system for PIFF these were assigned a unique class for analysis. Surgical methods were obtained as specific treatment codes according to Nordic Medico‐Statistical Committee (NOMESCO) and then sorted into more generalized treatment groups (REF).

#### Time to surgery

Timestamps from the time of radiology and the start of surgery were used to calculate the time to surgery in hours. Time of radiology was used as the baseline.

#### Outcome variables

The definition of an AE was peri‐ and postoperative events within 90 days postop. All‐cause AE data were used due to the nature of available data, AE include complications that occur during the surgical procedure, such as excessive bleeding, anaesthesia‐related complications or intraoperative fractures, and postoperative events such as infections, thromboembolic events, or mechanical complications. Preventability and accountability were represented using the Likert scale and complemented with the national coordinating council for medication error reporting and prevention (NCC‐MERP) index. To later facilitate analysis, the Likert scales were reduced into smaller, more easily comparable groups, this was also applied to the NCC‐MERP scale (Table 2).

**Table 2:** Classification of AEs according to preventability, accountability, and NCC‐MERP.

Preventability	Accountability	NCC‐MERP
1‐ Harm was not prevented	1‐ Harm was not caused by health care	E‐ Event contributing to or resulting in temporary harm
2‐ Harm was probably not preventable	2‐ Harm was probably not caused by health care	F‐ Event contributing to or resulting in temporary harm requiring outpatient care, inpatient care or prolonged hospital stay
3‐ Harm was probably preventable	3‐ Harm was probably caused by health care	G‐ Event contributing to or resulting in permanent harm
4‐ Harm was preventable	4‐ Harm was caused by health care	H‐ Event requiring life support within 60 min
		I‐ Event contributing to patient death

Abbreviations: AE, adverse event; NCC‐MERP, national coordinating council for medication error reporting and prevention.

Mortality was defined as the death of a patient, due to any cause within 90 days postsurgery, mortality includes both in‐hospital and out‐of‐hospital deaths. All‐cause mortality was used due to the nature of available data. Readmission was defined to be the admission of patients to inpatient care at any point within 90 days postoperatively where the cause for readmission could not be misattributed to the surgery. Exacerbations of existing conditions or infections assessed to be noncommunity acquired were included as viable reasons for readmissions within the scope of the study. Reoperations were defined as any surgical procedure related to the initial surgery or its complications that occurred within the first 90‐day postsurgery, these include all surgical complications such as hardware failure, infection requiring surgical debridement, or correction of surgical complications.

### Statistical analysis

Linear regression analysis was used for the dependent continuous variable. Binary logistic regression was used for odds ratios for the incidence of any AE with respect to group, time to surgery, CFS, BMI, perioperative bleeding, cognitive impairment (Yes or No), and ASA‐class (1&2 or 3&4). Ordinal variables with skewed distribution as determined by the Kolmogorov–Smirnov test were analysed using the Mann–Whitney *U* test. Comparisons between cases and controls for categorical data such as death and the incidence of any AE were made using the two‐sided Fisher's exact test. A *p* value of ≤0.05 was considered statistically significant. All analysis was performed using IBM SPSS Statistics (Version 24.1).

## RESULTS

**Figure 1:**
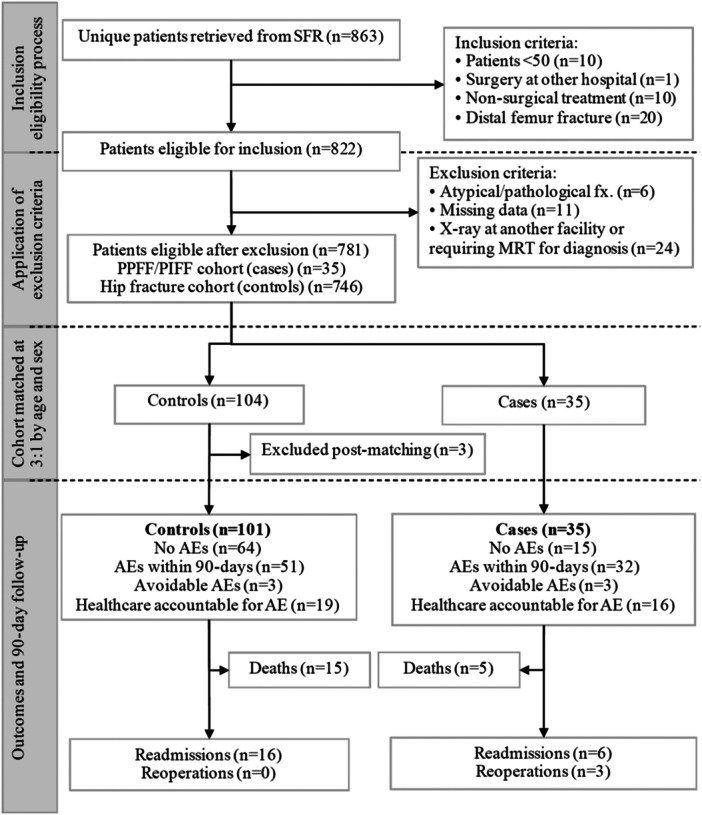
Flowchart for case and control inclusion.

The study sample consisted of 136 participants, 35 cases and 101 controls, obtained from a total of 863 hip fracture patients admitted to Danderyd hospital during 2020 (Figure 1).

### Descriptive statistics

The total patient cohort was 76.5% female with a mean age of 82 years at admission and identical age ranges within the groups. Mean clinical frailty scale was 4.47. Dementia and cognitive impairment showed no difference between the groups *p* = 0.688 (Table 3).

**Table 3:** Patient statistics and analysis for the case and control groups matched with respect to age and sex.

	Controls (*n* = 101)	Cases (*n* = 35)	Total (*n* = 136)	*p* Value
Patient characteristics				
Sex, *n* (%)				
Male	24 (23.8)	8 (22.9)	32 (23.5)	
Female	77 (76.2)	27 (77.1)	104 (76.5)	
Age, y, mean (min–max)	82.27 (60–97)	82.14 (60–97)	82.24 (60–97)	
ASA,^a^ *n* (%)				0.810
1	2 (2.0)	1 (2.9)	3 (2.2)	
2	29 (28.7)	11 (31.4)	40 (29.2)	
3	64 (62.4)	20 (57.1)	84 (61.3)	
4	7 (6.9)	3 (8.6)	10 (7.3)	
5	0 (0)	0 (0)	0 (0)	
Mean	2.74	2.71	2.74	
BM**I**,^b^ mean (min–max)	23.9 (15–45)	24.5 (17–32)	24.1 (15‐45)	0.528
Clinical Frailty Scale,^a^ mean (median)	4.48 (5)	4.37 (5)	4.45 (5)	0.581
Dementia or cognitive impairment,^a^ *n* (%)				0.688
Yes	25 (24.8)	8 (22.9)	33 (24.3)	
No	68 (67.3)	23 (65.7)	91 (66.9)	
Undergoing investigation	5 (5)	3 (8.6)	8 (5.9)	
Not reported	3 (3)	1(2.9)	4 (2.9)	

Abbreviations: ASA, American Society of Anaesthesiologists; BMI, body mass index.

^a^ Mann–Whitney *U* test.

^b^ Linear regression.

Perioperative bleeding was higher in cases compared with controls, *p* < 0.001. The distribution of anaesthesiological methods differed between the groups with most controls receiving spinal anaesthesia while cases received mostly spinal and epidural analgesia (EDA) combined. Most controls received either cephalomedullary nails (44%) or a prosthetic (36%) while cases received a plate fixation (57.1%) or prosthetic (20%) and in two cases fixation with combined surgical methods. The only cases to receive cephalomedullary nails as a definitive treatment were PIFF (8.6%) (Table 4).

**Table 4:** Fracture and treatment characteristics.

	Controls (*n* = 101)	Cases (*n* = 35)	Total (*n* = 136)	*p* Value
Fracture characteristics				
UCS, *n* (%)				
A_G_		1 (2.86%)		
B1		6 (17.1%)		
B2		4 (11.4%)		
B3		10 (28.6%)		
C		8 (22.9%)		
PIFF		6 (17.1%)		
AO/OTA classification, *n* (%)				
MAO‐31‐A1 (simple pertrochanteric)	9 (8.91%)			
MAO‐31‐A2 (multifragment pertrochanteric)	41 (40.6%)			
MAO‐31‐A3 (intertrochanteric)	2 (1.98%)			
MAO‐31‐B1 (subcapital)	8 (7.92%)			
MAO‐31‐B2 (transcervical)	41 (40.6%)			
Treatment characteristics				
Perioperative bleeding,^a^ mL, mean (min–max)	185 (0–800)	448 (99–1105)	256 (0–1105)	<0.001
Anaesthesiological method, *n* (%)				
Spinal anaesthesia	86 (85.1)	2 (5.7)	88 (64.7)	
Spinal+EDA	1 (1)	31 (88.6)	32 (23.5)	
Regional/local anaesthesia	4 (4)	0 (0)	4 (2.9)	
General anaesthesia	10 (9.9)	2 (5.7)	12 (8.8)	
Surgical procedure				
Cephalomedullary nail	44 (44)	3 (8.6)	47 (34.8)	
Prosthetic	36 (36)	7 (20)	43 (31.9)	
Sliding hip screw	7 (7)	1 (2.9)	8 (5.9)	
Parallel hip screws/pins	12 (12)	0 (0)	12 (8.9)	
Plate fixation	0 (0)	20 (57.1)	20 (14.8)	
Screw fixation	1 (11)	0 (0)	1 (0.7)	
Other fracture fixation or combined method	0 (0)	2 (5.7)	2 (1.5)	
Other operation	0 (0)	2 (5.7)	2 (1.5)	

*Note*: Statistics and analysis pertaining subgroups and surgical treatments for the case and control groups.

Abbreviations: AO/OTA, AO Foundation and Orthopaedic Trauma Association; MAO, morphology of AO/OTA classification; PIFF, peri‐implant femoral fracture; UCS, Unified Classification System.

^a^ Linear regression.

### Main results

**Figure 2:**
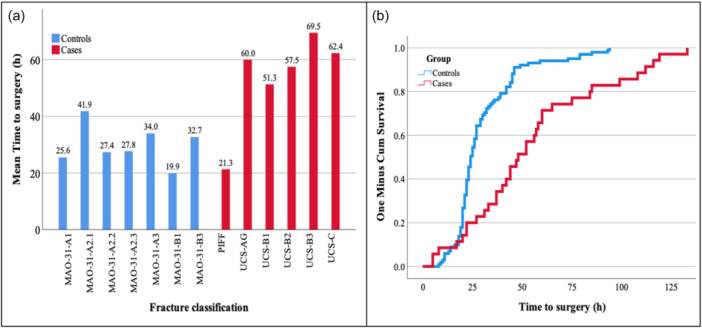
Mean time to surgery within the fracture subgroups groups (a). Kaplan–Meier time plot of time to surgery versus survival for cases and controls (b).

The mean time for cases from radiology to surgery was 54.9 h with controls being significantly faster at 30.5 h, *p* < 0.001 (Table 5). UCS‐B3 fractures had the longest time to surgery at almost 70 h (Figure 2a). The difference in time to surgery within the case‐subgroups to be borderline significant, *p* = 0.068. Ninety‐day mortality did not differ between the groups, *p* = 0.589, neither did time to death, *p* = 0.541 (Figure 2b). Controls were observed to have a greater range of time to death at 70 days compared to the cases at 34 days.

**Table 5:** Outcome statistics and analysis for the case and control groups matched with respect to age and sex.

	Controls (*n* = 101)	Cases (*n* = 35)	Total (*n* = 136)	*p* Value
Main results				
Time to surgery,^a^ h, mean (min–max)	30.1(8–94)	54.9 (5–133)	36.5 (5–133)	**<0.001**
Deaths				
Within 90‐days,^b^ *n* (%)	15 (14.9)	5 (14.3)	20 (14.7)	0.589
Time to death,^a^ d, mean (min–max)	32.5 (3–73)	22.2 (6–37)	29.9 (3–73)	0.541
Adverse events				
Patients with an AE,^b^ *n* (%)	37 (36.6)	20 (57.1)	57 (41.9)	**0.046**
Preventable, n	3	3	6	
Healthcare accountable, *n*	19	16	35	
NCC‐MERP: no harm, n	2	4	6	
NCC‐MERP: E–F, *n*	37	20	57	
NCC‐MERP: G–I, *n*	12	8	20	
Readmissions,				
Within 90 days,^c^ *n* (%)	16 (15.8)	6 (17.1)	22 (16.2)	0.858
Time to readmission,^a^ d, mean (min–max)	36.4 (8–70)	47.2 (6–86)	39.3 (6–86)	0.768
Reoperations				
Within 90 days, *n* (%)	0 (0)	3 (8.6)	3 (8.6)	
Time to reoperation, d, mean (min–max)	‐	2.33 (0–5)	2.33 (0–5)	

*Note*: Significant *p* values are marked in bold.

Abbreviations: AE, adverse event; NCC‐MERP, national coordinating council for medication error reporting and prevention.

^a^ Linear regression.

^b^ Fisher's exact test.

^c^ Pearson's *χ* ^2^ test.

### AEs

There was a total of 83 AEs with 20 cases being subjected to 32 AEs and 37 controls experienced 51 AEs. Cases were significantly more prone to AE compared with controls, *p* = 0.046. Six AEs were determined to be preventable while health care was deemed to be.

There was a borderline significant increase in AEs amongst cases compared with controls, with adjusted odds ratios of 2.751 (95% confidence interval [CI]: 0.92–8.25, *p* = 0.071). Additionally, there is a trend towards higher odds of AEs amongst patients with ASA scores of 3&4 compared with 1&2, with adjusted odds ratios of 2.530 (95% CI: 0.99–6.46, *p* = 0.052) (Table 6).

**Table 6:** Odds ratios (OR) for the incidence of any adverse event depended on selected descriptive and outcome variables.

Variable^a^	Unadjusted			Adjusted		
	OR	95% CI	*p* Value	OR	95% CI	*p* Value
Group						
Controls (Ref.)	1			1		
Cases	2.306	1.06–5.04	**0.036**	2.751	0.92–8.25	0.071
Cognitive impairment						
No (Ref.)	1			1		
Yes	1.2	0.55–2.67	0.636	1.008	0.4–2.53	0.987
Time to surgery	1.013	1–1.03	0.075	1.006	0.99–1.02	0.494
CFS	1.074	0.89–1.3	0.470	1.069	0.84–1.35	0.580
BMI	0.988	0.91–1.07	0.763	0.974	0.89–1.07	0.579
Bleeding	1	1–1.002	0.765	1	1–1.001	0.567
ASA 1&2 (Ref.)	1					
ASA 3&4	2.072	0.96–4.47	0.063	2.530	0.99–6.46	0.052

Abbreviations: ASA, American Society of Anaesthesiologists; BMI, body mass index; CI, confidence interval; CFS, Clinical Frailty Scale.

^a^ Binary logistic regression.

## DISCUSSION

This retrospective matched cohort study examined the difference in delay to surgery for hip fracture and PPFF/PIFF. There was a significant difference in the time to surgery from radiology, as well as the incidence of any AE in an unadjusted analysis between standard hip fracture patients and PPFF/PIFF patients. Mortality and readmissions between the cases and controls did not show a significant difference.

Time to surgery is an important measure in hip fractures. Hip fracture accelerated surgical treatment and care track (HIP ATTACK), a randomized controlled trial comparing accelerated surgical intervention to standard care found that intervention time for standard care was 10–42 h from diagnosis, with the accelerated path resulting in surgery within 6 h [11]. The results of the HIP ATTACK trial do not contradict the existing consensus that surgery within 48 h decreases mortality. However, it brings to question the need for surgery within 6 h from diagnosis, as there was no apparent benefit to mortality or other serious complications represented by a composite outcome.

We found that there was a significant difference in time to surgery between the cases and controls, a novel observation. Furthermore, the time to surgery within the subgroups saw a fairly even distribution between the hip fracture classes with multifragmentary pertrochanteric fractures being the most delayed to surgery and transcervical fractures amongst the least delayed. Observing the subgroups within the case cohort, PIFFs being notably faster to receive surgical intervention compared with the PPFF subgroups. The mean time to surgery for PPFFs has been reported within the ranges 31.2–105 h [7], placing the observed population into the middle tier of delay at 54.9 h. It is worth noting that there is inconsistency in data collection in the literature for time to surgery for PPFF as studies have used varying measures, such as time of admission or time of diagnosis rather than time from radiology [1, 13].

The increase in delay to surgery for PPFF is most likely in part due to the complicated circumstances such as poor bone quality, lack of appropriate materials, and complex fracture patterns requiring greater expertise from the surgeon. Studies have yielded different conclusions regarding the significance of such delays. Griffiths et al. observed a delay to surgery >72 h to be significant for an increased risk of an AE [8] similar to that of other findings associating a delay >24 h with increased morbidity [1]. Studies conducted by Johnson‐Lynn et al. and Sellan et al. resulted in no significant difference in mortality or postoperative complications, with the prior making the argument that the greater delay to surgery allows for the acquisition of necessary materials and competence to perform a complex surgery [12, 21].

There was no significant difference regarding mortality or readmissions between groups in our study. This is in line with previous studies which show little or no correlation between delay to surgery and mortality in PPFF [12, 21]. However, the impact of time to surgery on the incidence of any AE when unadjusted was borderline significant at *p* = 0.075. As previously described by Kelly‐Pettersson et al., delay in surgery and belonging to a higher ASA class are prominent risk factors in increasing the rate of AEs in hip fracture patients [18]. Furthermore, various investigations have explored the rate of AEs in PPFFs and reaching varying conclusions. Further investigations to establish the parallel between morbidity and PPFFs are warranted.

In our study, the significant delay in time to surgery observed in patients with periprosthetic and PIFFs compared with standard hip fractures has important clinical implications. Delays in surgical intervention may contribute to prolonged pain, decreased mobility, and increased hospital stays, which can negatively impact patient outcomes and overall quality of life, in particular amongst the elderly. The higher incidence of AEs in the delayed surgery group suggests that timely intervention is critical in preventing complications such as deep vein thrombosis, pulmonary complications, and pressure ulcers, which are common in immobilized patients. These findings highlight the need for revisiting and potentially revising hospital protocols to prioritize surgical interventions for PPFF and PIFF cases, similar to the fast‐track systems in place for standard hip fractures. Integrating these cases into fast‐track protocols could reduce the observed delays and improve patient outcomes.

PPFFs are considered a growing issue, as we observe an ageing population and increasing numbers of THAs performed each year [2]. Comparing PPFFs to standard hip fractures that undergo the fast‐track highlights the potential displacement effect of these patients. A recent study by Kruse et al. found 41 PIFFs in a population of 1965 Swedish hip fracture patients ≥50 years of age, and a 1‐year mortality of 34% following fracture [17]. Kruse made the argument that the incidence of PIFFs may come to increase, as the elderly population increase in size. However, a difficulty in studying PPFF is the lack of a UCS. PIFFs do not currently have a generally accepted classification system. New classification systems have been proposed in attempts to standardize research within the field. Current proposals by various groups are taking into consideration factors such as fracture morphology, type of implant, and healing which might hopefully aid in studying this patient population [27, 28].

All patients during the time period of the study were eligible for inclusion, thus limiting inherent selection bias. The limitations of the study include the data sources, as SFR was founded recently, and input to its database was only introduced to Danderyd hospital as of the end of 2019 as well as the unicenter study design. To validate the results of our study and further explore differences within this patient population, a greater sample size of PIFFs and PPFF subgroups would have to be included in the analysis. We suggest that an appropriate follow‐up study would take the form of a randomized controlled trial similar to HIP‐ATTACK, where patients, depending on their period of inclusion, receive either standard care or undergo a fast‐track. Socioeconomic factors such as migrant status, mean income, and level of highest education are associated with increased mortality and readmission for hip fracture patients, such factors should therefore be considered in follow‐up studies [16]. Given the limitations of our single‐center design and relatively small sample size, future studies should consider a multicenter approach to increase the generalizability of results. A prospective cohort study design would allow for real‐time data collection while enhancing the accuracy of findings related to surgical timing and outcomes. Larger studies would also allow for more detailed subgroup analyses, which are critical to explore the impact of specific fracture types, surgical methods, and comorbidities on surgical timing and outcomes. We also recommend that future studies incorporate more detailed comorbidity data, such as the Elixhauser Comorbidity Index, and consider including patient‐reported outcome measures to capture the patient's perspective on their recovery.

## CONCLUSION

This study reveals a significant delay in surgery for PPFFs and PIFFs compared with standard hip fractures, suggesting higher AE rates. While mortality and readmissions did not differ significantly, the delay underscores the need for timely intervention in these complex cases. Further research is needed to address these challenges and improve patient outcomes.

## AUTHOR CONTRIBUTIONS

**Olof Sköldenberg, Martin Magnéli**: Conceptualization. **Olof Sköldenberg, Martin Magnéli**: Methodology. **Felix Alarcón, Olof Sköldenberg, Martin Magnéli, Michael Axenhus**: Software. **Olof Sköldenberg, Martin Magnéli, Michael Axenhus**: Validation. **Felix Alarcón, Martin Magnéli, Michael Axenhus**: Formal analysis. **Felix Alarcón, Michael Axenhus, Martin Magnéli**: Investigation. **Olof Sköldenberg, Martin Magnéli, Michael Axenhus**: Resources. **Olof Sköldenberg, Martin Magnéli, Michael Axenhus**: Data curation. **Felix Alarcón**: Writing—original draft. **Olof Sköldenberg, Martin Magnéli, Michael Axenhus**: Writing—review & editing. **Felix Alarcón**: Visualization. **Olof Sköldenberg, Martin Magnéli**: Supervision.

## CONFLICT OF INTEREST STATEMENT

The authors declare no conflict of interest.

## ETHICS STATEMENT

This study was conducted in accordance with the Helsinki Declaration and was approved by the Ethics Committee of Karolinska Institutet (entry number dnr 2013/238‐31/2). According to the ethical permit, individual consent was not needed in this observational cohort.

## Data Availability

The data are available from the corresponding author on reasonable request.

## References

[1] Boddapati, V. , Grosso, M.J. , Sarpong, N.O. , Geller, J.A. , Cooper, H.J. & Shah, R.P. (2019) Early morbidity but not mortality increases with surgery delayed greater than 24 hours in patients with a periprosthetic fracture of the hip. The Journal of Arthroplasty, 34(11), 2789–2792.e1. Available from: 10.1016/j.arth.2019.06.027 31279604

[2] Chatziagorou, G. , Lindahl, H. , Garellick, G. & Kärrholm, J. (2019) Incidence and demographics of 1751 surgically treated periprosthetic femoral fractures around a primary hip prosthesis. HIP International, 29(3), 282–288. Available from: 10.1177/1120700018779558 30009622

[3] Cheng, S.Y. , Levy, A.R. , Lefaivre, K.A. , Guy, P. , Kuramoto, L. & Sobolev, B. (2011) Geographic trends in incidence of hip fractures: a comprehensive literature review. Osteoporosis International, 22(10), 2575–2586. Available from: 10.1007/s00198-011-1596-z 21484361

[4] Court‐Brown, C.M. & Caesar, B. (2006) Epidemiology of adult fractures: a review. Injury(), 37(8), 691–697. Available from: 10.1016/j.injury.2006.04.130 16814787

[5] Dhanwal, D.K. , Dennison, E.M. , Harvey, N.C. & Cooper, C. (2011) Epidemiology of hip fracture: worldwide geographic variation. Indian Journal of Orthopaedics, 45(1), 15–22. Available from: 10.4103/0019-5413.73656 21221218 PMC3004072

[6] Eriksson, M. , Kelly‐Pettersson, P. , Stark, A. , Ekman, A.K. & Sköldenberg O. (2012) Straight to bed' for hip‐fracture patients. Injury(), 43(12), 2126–2131. Available from: 10.1016/j.injury.2012.05.017 22769975

[7] Farrow, L. , Ablett, A.D. , Sargeant, H.W. , Smith, T.O. & Johnston, A.T. (2021) Does early surgery improve outcomes for periprosthetic fractures of the hip and knee? A systematic review and meta‐analysis. Archives of Orthopaedic and Trauma Surgery, 141(8), 1393–1400. Available from: 10.1007/s00402-020-03739-2 33555402 PMC8295128

[8] Griffiths, E.J. , Cash, D.J.W. , Kalra, S. & Hopgood, P.J. (2013) Time to surgery and 30‐day morbidity and mortality of periprosthetic hip fractures. Injury, 44(12), 1949–1952. Available from: 10.1016/j.injury.2013.03.008 23639824

[9] Harris, P.A. , Taylor, R. , Thielke, R. , Payne, J. , Gonzalez, N. & Conde, J.G. (2009) Research electronic data capture (REDCap)‐‐a metadata‐driven methodology and workflow process for providing translational research informatics support. Journal of Biomedical Informatics, 42(2), 377–381. Available from: 10.1016/j.jbi.2008.08.010 18929686 PMC2700030

[10] Haughom, B.D. , Basques, B.A. , Hellman, M.D. , Brown, N.M. , Della Valle, C.J. & Levine, B.R. (2018) Do mortality and complication rates differ between periprosthetic and native hip fractures? The Journal of Arthroplasty, 33(6), 1914–1918. Available from: 10.1016/j.arth.2018.01.046 29526336

[11] Borges, F.K. , Bhandari, M. , Guerra‐Farfan, E. , Patel, A. , Sigamani, A. , Umer, M. et al. (2020) Accelerated surgery versus standard care in hip fracture (HIP ATTACK): an international, randomised, controlled trial. The Lancet, 395(10225), 698–708. Available from: 10.1016/S0140-6736(20)30058-1 32050090

[12] Johnson‐Lynn, S. , Ngu, A. , Holland, J. , Carluke, I. & Fearon, P. (2016) The effect of delay to surgery on morbidity, mortality and length of stay following periprosthetic fracture around the hip. Injury, 47(3), 725–727. Available from: 10.1016/j.injury.2015.11.013 26653266

[13] Jones, C.S. , Eardley, W.G.P. , Johansen, A. , Inman, D.S. & Evans, J.T. (2023) Caring for patients with periprosthetic femoral fractures across England and Wales in 2021. Bone & Joint Open, 4(5), 378–384. Available from: 10.1302/2633-1462.45.BJO-2023-0011.R1 37219370 PMC10204652

[14] Kelly‐Pettersson, P. , Samuelsson, B. , Muren, O. , Unbeck, M. , Gordon, M. , Stark, A. et al. (2017) Waiting time to surgery is correlated with an increased risk of serious adverse events during hospital stay in patients with hip‐fracture: a cohort study. International Journal of Nursing Studies, 69, 91–97. Available from: 10.1016/j.ijnurstu.2017.02.003 28189926

[15] Klestil, T. , Röder, C. , Stotter, C. , Winkler, B. , Nehrer, S. , Lutz, M. et al. (2018) Impact of timing of surgery in elderly hip fracture patients: a systematic review and meta‐analysis. Scientific Reports, 8(1). 13933. Available from: 10.1038/s41598-018-32098-7 30224765 PMC6141544

[16] Kristensen, P.K. , Thillemann, T.M. , Pedersen, A.B. , Søballe, K. & Johnsen, S.P. (2017) Socioeconomic inequality in clinical outcome among hip fracture patients: a nationwide cohort study. Osteoporosis International, 28(4), 1233–1243. Available from: 10.1007/s00198-016-3853-7 27909785

[17] Kruse, M. , Mohammed, J. , Sayed‐Noor, A. , Wolf, O. , Holmgren, G. , Nordström, R. et al. (2022) Peri‐implant femoral fractures in hip fracture patients treated with osteosynthesis: a retrospective cohort study of 1965 patients. European Journal of Trauma and Emergency Surgery, 48(1), 293–298. Available from: 10.1007/s00068-020-01596-7 33484277

[18] LeBlanc, K.E. , Muncie HL, J.r & LeBlanc, L.L. (2014) Hip fracture: diagnosis, treatment, and secondary prevention. American Family Physician, 89(12), 945–951. 25162161

[19] Möller, M. , Wolf, O. , Bergdahl, C. , Mukka, S. , Rydberg, E.M. , Hailer, N.P. et al. (2022) The Swedish Fracture Register—ten years of experience and 600,000 fractures collected in a National Quality Register. BMC Musculoskeletal Disorders, 23(1), 141. Available from: 10.1186/s12891-022-05062-w 35148730 PMC8832767

[20] Ramavath, A. , Lamb, J.N. , Palan, J. , Pandit, H.G. & Jain, S. (2020) Postoperative periprosthetic femoral fracture around total hip replacements: current concepts and clinical outcomes. EFORT Open Reviews, 5(9), 558–567. Available from: 10.1302/2058-5241.5.200003 33072408 PMC7528669

[21] Sellan, M.E. , Lanting, B.A. , Schemitsch, E.H. , MacDonald, S.J. , Vasarhelyi, E.M. & Howard, J.L. (2018) Does time to surgery affect outcomes for periprosthetic femur fractures? The Journal of Arthroplasty, 33(3), 878–881. Available from: 10.1016/j.arth.2017.10.045 29174404

[22] Simunovic, N. , Devereaux, P.J. , Sprague, S. , Guyatt, G.H. , Schemitsch, E. , Debeer, J. et al. (2010) Effect of early surgery after hip fracture on mortality and complications: systematic review and meta‐analysis. Canadian Medical Association Journal, 182(15), 1609–1616. Available from: 10.1503/cmaj.092220 20837683 PMC2952007

[23] Singh, J.A. , Jensen, M.R. , Harmsen, S.W. & Lewallen, D.G. (2013) Are gender, comorbidity, and obesity risk factors for postoperative periprosthetic fractures after primary total hip arthroplasty? The Journal of Arthroplasty, 28(1), 126–131.e2. Available from: 10.1016/j.arth.2012.03.010 22552223 PMC3414633

[24] Singh, J.A. & Lewallen, D.G. (2012) Peptic ulcer disease and heart disease are associated with periprosthetic fractures after total hip replacement. Acta Orthopaedica, 83(4), 353–359. Available from: 10.3109/17453674.2012.717844 22900908 PMC3427625

[25] Streubel, P. (2013) Mortality after periprosthetic femur fractures. Journal of Knee Surgery, 26(1), 027–030. Available from: 10.1055/s-0033-1333905 23393056

[26] Swiontkowski, M.F. , Agel, J. , McAndrew, M.P. , Burgess, A.R. & MacKenzie, E.J. (2000) Outcome validation of the AO/OTA fracture classification system. Journal of Orthopaedic Trauma, 14(8), 534–541. Available from: 10.1097/00005131-200011000-00003 11149498

[27] Toro, G. , Moretti, A. , Ambrosio, D. , Pezzella, R. , De Cicco, A. , Landi, G. et al. (2021) Fractures around trochanteric nails: the “vergilius classification system. Advances in Orthopedics, 2021. 7532583. Available from: 10.1155/2021/7532583 33520318 PMC7817309

[28] Videla‐Cés, M. , Sales‐Pérez, J.‐M. , Sánchez‐Navés, R. , Romero‐Pijoan, E. & Videla, S. , Peri‐implant Femoral Fractures Study Group (2019) Proposal for the classification of peri‐implant femoral fractures: Retrospective cohort study. Injury, 50(3), 758–763.Available from: 10.1016/j.injury.2018.10.042 30424840

